# First Annotated Genome of a Mandibulate Moth, *Neomicropteryx cornuta*, Generated Using PacBio HiFi Sequencing

**DOI:** 10.1093/gbe/evab229

**Published:** 2021-10-01

**Authors:** Xuankun Li, Emily Ellis, David Plotkin, Yume Imada, Masaya Yago, Jacqueline Heckenhauer, Timothy P Cleland, Rebecca B Dikow, Torsten Dikow, Caroline G Storer, Akito Y Kawahara, Paul B Frandsen

**Affiliations:** 1 McGuire Center for Lepidoptera and Biodiversity, Florida Museum of Natural History, University of Florida, USA; 2 Graduate School of Science and Engineering, Ehime University, Matsuyama, Japan; 3 The University Museum, The University of Tokyo, Hongo, Bunkyo-ku, Japan; 4 LOEWE Centre for Translational Biodiversity Genomics (LOEWE-TBG), Frankfurt, Germany; 5 Department of Terrestrial Zoology, Entomology III, Senckenberg Research Institute and Natural History Museum Frankfurt, Frankfurt, Germany; 6 Museum Conservation Institute, Smithsonian Institution, Suitland, Maryland, USA; 7 Data Science Lab, Office of the Chief Information Officer, Smithsonian Institution, Washington, District of Columbia, USA; 8 Department of Entomology, National Museum of Natural History (USNM), Smithsonian Institution, Washington, District of Columbia, USA; 9 Department of Plant and Wildlife Sciences, Brigham Young University, USA

**Keywords:** Amphiesmenoptera, HiFi, IsoSeq, Micropterigidae, Lepidoptera, PacBio

## Abstract

We provide a new, annotated genome assembly of *Neomicropteryx cornuta*, a species of the so-called mandibulate archaic moths (Lepidoptera: Micropterigidae). These moths belong to a lineage that is thought to have split from all other Lepidoptera more than 300 Ma and are consequently vital to understanding the early evolution of superorder Amphiesmenoptera, which contains the order Lepidoptera (butterflies and moths) and its sister order Trichoptera (caddisflies). Using PacBio HiFi sequencing reads, we assembled a highly contiguous genome with a contig N50 of nearly 17 Mb. The assembled genome length of 541,115,538 bp is about half the length of the largest published Amphiesmenoptera genome (*Limnephilus lunatus*, Trichoptera) and double the length of the smallest (*Papilio polytes*, Lepidoptera). We find high recovery of universal single copy orthologs with 98.1% of BUSCO genes present and provide a genome annotation of 15,643 genes aided by resolved isoforms from PacBio IsoSeq data. This high-quality genome assembly provides an important resource for studying ecological and evolutionary transitions in the early evolution of Amphiesmenoptera.

**Significance**

*Neomicropteryx cornuta* is a member of the family Micropterigidae, sister to all other extant Lepidoptera. In this article, we report the first high-quality genome of a micropterigid, which is essential for studying ecological and evolutionary transitions in the early evolution of superorder Amphiesmenoptera.

## Introduction

Lepidoptera are one of the most diverse herbivorous insect lineages, with more than 160,000 described species (Mitter et al. 2017). They are one of the two extant orders, along with caddisflies (Trichoptera), that comprise the superorder Amphiesmenoptera. Modern Lepidoptera and Trichoptera are morphologically similar in many ways, including dense covering of hairs or scales on their wings (Kristensen 1984), but in the ∼310 Myr since they were believed to have diverged from each other (Kawahara et al. 2019), they have developed very different behaviors and ecological roles. Trichoptera larvae are primarily aquatic, with a diversity of feeding behaviors ranging from pure herbivory to opportunistic scavenging to predation (Mackay and Wiggins 1979). In contrast, almost all Lepidoptera larvae are terrestrial and herbivorous, and the adults provide essential pollination services for many flowering plants (Scoble 1992). 

The earliest diverging lineage of Lepidoptera includes the mandibulate archaic moths, Micropterigidae (Kawahara et al. 2019). The family includes roughly 20 extant genera (Van Nieukerken et al. 2011), and is sister to all other extant Lepidoptera. Its fossil record dates back to the Lower Cretaceous (Azar et al. 2010; Kristensen and Skalski 1999; Whalley 1978), though recent phylogenetic studies estimate that the family could be as old as 300 Myr (Kawahara et al. 2019). Micropterigids are known for their unusual feeding habits and mouthpart morphology relative to other moths. The larvae feed on liverworts (Imada et al. 2011), whereas the larvae of most other extant Lepidoptera feed on angiosperms. Many micropterigid larvae, including those in the genus *Neomicropteryx*, have a plastron and other morphological features conducive to survival in flooded habitats (Davis and Landry 2012); this aquatic association is in sharp contrast with the primarily terrestrial habitats of most other Lepidoptera. Micropterigid adults have mandibulate (chewing) mouthparts, with some species feeding on angiospermous pollen (Kristensen 1999) or spores of ferns and lycopods (Gibbs 2014), whereas most other adult Lepidoptera are either nonfeeding or consume nectar with an elongate, flexible proboscis (siphoning-sucking mouthparts). Fossil evidence suggests that the most recent common ancestor of Lepidoptera was mandibulate, like extant Micropterigidae, and had small structures called galea (also present in micropterigids) that evolved into the proboscis found in nearly all other butterflies and moths (Kristensen 1984; Krenn 2010). Since micropterigids remained mandibulate, their genetic makeup could shed light on the early evolution of ancient Lepidoptera and Trichoptera.

Despite their unique ecology and the fact that Micropterigidae represent a possible important transition between Trichoptera and Lepidoptera, there are no existing genome assemblies of Micropterigidae. By November 2020, there were 118 Lepidoptera and six Trichoptera genome assemblies available on GenBank (Hotaling et al. 2021). With more than 250 Myr of evolution between available genomes of Trichoptera and Lepidoptera (Triant et al. 2018), a Micropterigidae genome is an important evolutionary resource. Moreover, both orders are known for producing silk, but the structure and function of that silk can vary greatly between the two orders. Modern genomic analysis is an essential tool for extrapolating the evolutionary processes and transitions that resulted in the extant diversity stemming from the ancestral amphiesmenopteran. Here, we provide an annotated genome of *Neomicropteryx cornuta*, the first available genome of any mandibulate Lepidoptera.

We use PacBio HiFi sequencing data to assemble a highly contiguous *N.cornuta* genome. This is especially important since many genomes of Lepidoptera are of low quality (Ellis et al. 2021). We also provide a genome annotation by resolved isoforms from PacBio IsoSeq data. Our genome assembly provides an important resource to study ecological and evolutionary transitions in the early evolution of Amphiesmenoptera and sets the stage for future studies on the genomics of Amphiesmenoptera.

## Results and Discussion

### Assembly

**Fig. 1.**
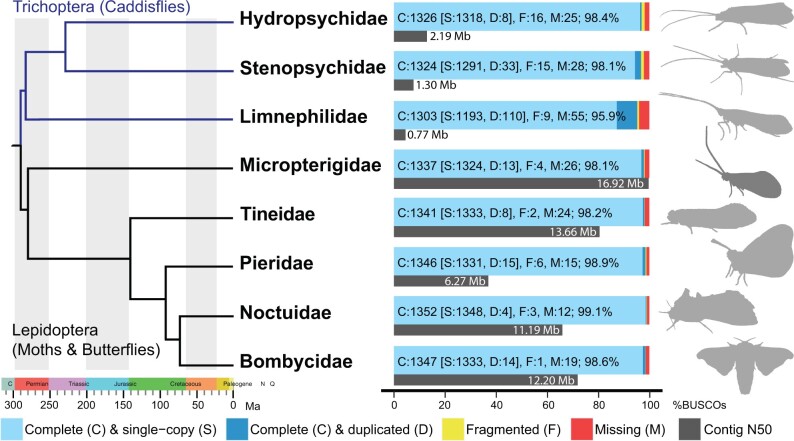
Comparison of BUSCO (blue, yellow and red bars) and contig N50 (gray bars) results for the genomes of Trichoptera and Lepidoptera species (from top to bottom: *Hydropsyche tenuis* (Hydropsychidae), *Stenopsyche tienmushanensis* (Stenopsychidae), *Hesperophylax magnus* (Limnephilidae), *Neomicropteryx cornuta* (Micropterigidae), *Tinea trinotella* (Tineidae), *Anthocharis cardamines* (Pieridae), *Autographa gamma* (Noctuidae), *Bombyx mori* (Bombycidae)). Each silhouette to the right of the plots is derived from a member of the same genus as the genome assemblies. The phylogeny is based on Kawahara et al. (2019) and Thomas et al. (2020). Original photographs for silhouettes *T. trinotella* provided by Donald Hobern, *Au. Gamma* provide by Martin Olofsson; other silhouettes made from photographs by authors or drew by XL.

Sequencing the *N. cornuta* genome using two PacBio SMRT cells produced 8.8 and 8.4 Gb of HiFi data, respectively, corresponding to ∼31× PacBio HiFi read coverage. Blobtools analysis assigned 99.8% of all base pairs to the phylum Arthropoda (supplementary fig. 1, Supplementary Material online) and the resulting assembly contained 101 contigs with a contig N50 of 16,921,359 bp. This is the second-longest contig N50 for an amphiesmenopteran genome published thus far (Hotaling et al. 2021), shorter only than the genome of the silk moth *Samia ricini* (GCA_014132275.1), which was also generated by PacBio HiFi sequencing. Assembly GC content was 33.4% and the total assembly length was 541,115,538 bp, which is intermediate in length compared with other Amphiesmenoptera genomes (with BUSCO scores > 90%), which range from 227,005,758 bp (*Papilio polytes*) to 1,369,180,260 bp (*Limnephilus lunatus*) (Hotaling et al. 2021). BUSCO analysis identified 98.1% (97.9% complete; 0.3% fragmented) of the Insecta gene set in the assembly (fig. 1, table 1).

**Table 1:** Comparison of New *Neomicropteryx cornuta* Genome Assemblies Against Previously Published Representative Genomes of Amphiesmenoptera

Order	Family	Species	Source	Accession	Assembly Length (Mb)	Contig N50 (Mb)	BUSCOs Present (C%)
Lepidoptera	Micropterigidae	*Neomicropteryx cornuta*	Present study	JAHKQU000000000	541.12	16.92	98.1
Lepidoptera	Tineidae	*Tinea trinotella*	Wellcome Sanger Institute	GCA_905220615.1	371.74	13.66	98.2
Lepidoptera	Pieridae	*Anthocharis cardamines*	Wellcome Sanger Institute	GCA_905404175.1	359.62	6.27	98.9
Lepidoptera	Noctuidae	*Autographa gamma*	Wellcome Sanger Institute	GCA_905146925.1	373.07	11.19	99.1
Lepidoptera	Bombycidae	*Bombyx mori*	The University of Tokyo	GCA_014905235.2	460.35	12.20	98.6
Trichoptera	Limnephilidae	*Hesperophylax magnus*	Olsen et al. (2021)	JADDOG000000000	1233.59	0.77	95.9
Trichoptera	Hydropsychidae	*Hydropsyche tenuis*	Heckenhauer et al. (2019)	GCA_009617725.1	229.66	2.19	98.4
Trichoptera	Stenopsychidae	*Stenopsyche tienmushanensis*	Luo et al. (2018)	GCA_008973525.1	451.49	1.30	98.1

### Annotation

We also report the functional annotations of *N. cornuta*. Of the 15,643 predicted proteins, 86.62% (13,550) were verified by BLAST and/or transcript evidence, 63.04% (9,862) were mapped to GO terms, and 43.95% (6,875) were functionally annotated in Blast2Go. Top GO annotations include catalytic activity (4,512), cellular process (4,492), binding (4,414), and metabolic process (4,296) (supplementary figs. 4–6, Supplementary Material online). We annotated a total of 48.61% of the genome assembly as repeats using RepeatModeler and RepeatMasker. Unclassified repeats comprise >161 million bases, which is the highest among all types of repeats (genome proportion of 29.82%). Long interspersed nuclear elements (LINEs) are the second most abundant repeat category with >39 million bases (7.23%), followed by DNA transposons and rolling circles and long terminal repeats (LTRs), which have >15 million (2.82%) and >11 million bases (2.10%) respectively. Percent composition of repeats and predominance of LINEs were similar to both the *S.ricini* and *Bombyxmori* genome assemblies (Lee et al. 2021).

## Conclusions

Our results provide a new genome for a relict evolutionary lineage, separated by more than 250 Myr of evolution from any currently existing genome. Results from our study show that high fidelity, long-read sequencing facilitates the production of more contiguous assemblies and generates high-quality resources for further investigation of genome functions. Our new genome will be useful for future studies on amphiesmenopteran genetics, conservation and ecology.

## Materials and Methods

### Sequencing and Assembly

Larval specimens of *N. cornuta* were field collected at two sites in Kochi Prefecture, Japan, and flash frozen (supplementary note 1, Supplementary Material online). DNA was extracted from a single specimen using a Zymo Quick-prep HMW DNA extraction kit. Following DNA extraction, the sequencing library was prepared according to the “Using Express Template Prep Kit 2.0 With Low DNA Input” protocol from PacBio. The library was then sequenced on two PacBio Sequel II SMRT cells in CCS mode. Further details are provided in supplementary note 1, Supplementary Material online. Q20 HiFi CCS reads were generated from the raw data using the pbccs tool, which is included in the pbbioconda package (https://github.com/PacificBiosciences/pbbioconda, last accessed August 9, 2021). The reads were then assembled into contigs using Hifiasm v0.13-r307 with the option for aggressive duplicate purging enabled (option -l 2) (Cheng et al. 2021). The primary contig assembly was used for all downstream analyses.

RNA was extracted from the head and silk gland, and library preparation was performed using the IsoSeq express workflow. The library was then sequenced on a single Sequel II PacBio SMRT cell. Further details are provided in supplementary note 1, Supplementary Material online. The IsoSeq3 pipeline, part of the pbbioconda package, was used to generate IsoSeq clusters, following the published PacBio IsoSeq workflow (https://github.com/PacificBiosciences/IsoSeq/blob/master/isoseq-clustering.md, last accessed August 9, 2021). The steps in the pipeline are 1) circular consensus sequence calling (CCS read generation), 2) primer removal and demultiplexing, 3) refining (trimming of polyA tails and concatemer removal), 4) clustering, and 5) polishing.

We screened the genome assembly for potential contaminants with BlobTools v1.0 (Laetsch and Blaxter 2017) (supplementary note 2 and supplementary fig. 1, Supplementary Material online). We assessed genome quality and completeness with BUSCO v4.1.1 (Seppey et al. 2019) (supplementary note 3, Supplementary Material online) using the OrthoDB v.10 Insecta gene set (Kriventseva et al. 2019), and generated genome statistics using the assembly_stats v0.1.4 script (Trizna 2020) (supplementary table 1, Supplementary Material online, for full output). We conducted genome profiling (estimation of major genome characteristics such as size, heterozygosity, and repetitiveness) on the HiFi sequence data with GenomeScope 2.0 (Ranallo-Benavidez et al. 2020); these methods are described in supplementary note 4 and supplementary figures 3 and 4, Supplementary Material online.

### Repeat and Gene Annotation

We identified and classified repetitive elements de novo and generated a library of consensus repeat sequences for the genome using RepeatModeler 2.0 (Flynn et al. 2020). We then annotated and masked repeats in the assembly with RepeatMasker 4.1.1 (Smit et al. 2013–2015) using the custom repeat library generated in the previous step. Finally, we reran RepeatMasker on the masked genome using the Repbase arthropod repeat library (Bao et al. 2015). We annotated the *N. cornuta* genome assembly using MAKER v3.01.03 (Cantarel et al. 2008) and generated ab initio gene predictions using SNAP (Korf 2004), with more details provided in supplementary note 5, Supplementary Material online. To generate functional predictions on the predicted proteins, we used Blast2GO. First, we extracted the CDS sequences from the genome and then used blastx (nr, e-value 1e-4, max_target_seqs = 5) to compare the predicted genes against the NCBI RefSeq nonredundant protein database. We used Blast2GO v1.4.4 (Götz et al. 2008) to map functional annotation and GO terms to the resulting sequences.

## Supplementary Material

Supplementary data are available at *Genome Biology and Evolution* online.

## Supplementary Material

evab229_Supplementary_Data

## References

[evab229-B1] Azar D , Geze R, Acra F. 2010. Chapter 14 Lebanese Amber. In: Penny D, editor. Biodiversity of fossils in Amber form the major world deposits. Manchester (United Kingdom): Siri Scientific Press. p. 271–298.

[evab229-B2] Bao W , Kojima KK, Kohany O. 2015. Repbase update, a database of repetitive elements in eukaryotic genomes. Mob DNA. 6(1):11. 26045719 10.1186/s13100-015-0041-9 PMC4455052

[evab229-B3] Cantarel BL , et al 2008. MAKER: an easy-to-use annotation pipeline designed for emerging model organism genomes. Genome Res. 18(1):188–196. 18025269 10.1101/gr.6743907 PMC2134774

[evab229-B4] Cheng H , Concepcion GT, Feng X, Zhang H, Li H. 2021. Haplotype-resolved de novo assembly using phased assembly graphs with hifiasm. Nat Methods. 18(2):170–175. 33526886 10.1038/s41592-020-01056-5 PMC7961889

[evab229-B5] Davis DR , Landry JF. 2012. A review of the North American genus *Epimartyria* (Lepidoptera, Micropterigidae) with a discussion of the larval plastron. ZooKeys 183:37–83. 10.3897/zookeys.183.2556 PMC3332027 22573948

[evab229-B6] Ellis EA , Storer CG, Kawahara AY. 2021. De novo genome assemblies of butterflies. GigaScience 10(6):1–8. 10.1093/gigascience/giab041 PMC8170690 34076242

[evab229-B7] Flynn JM , et al 2020. RepeatModeler2 for automated genomic discovery of transposable element families. Proc Natl Acad Sci U S A. 117(17):9451–9457. 32300014 10.1073/pnas.1921046117 PMC7196820

[evab229-B8] Gibbs GW. 2014. Micropterigidae (Insecta: Lepidoptera). Fauna N. Z. 72:127.

[evab229-B9] Götz S , et al 2008. High-throughput functional annotation and data mining with the Blast2GO suite. Nucleic Acids Res. 36(10):3420–3435. 18445632 10.1093/nar/gkn176 PMC2425479

[evab229-B10] Heckenhauer J , et al 2019. Annotated draft genomes of two caddisfly species *Plectrocnemia conspersa* Curtis and *Hydropsyche tenuis* Navas (Insecta: Trichoptera). Genome Biol Evol. 11(12):3445–3451. 31774498 10.1093/gbe/evz264 PMC6916706

[evab229-B11] Hotaling S , et al 2021. Long-reads are revolutionizing 20 years of insect genome sequencing. Genome Biol Evol. 13:evab138. 34152413 10.1093/gbe/evab138 PMC8358217

[evab229-B12] Imada Y , Kawakita A, Kato M. 2011. Allopatric distribution and diversification without niche shift in a bryophyte-feeding basal moth lineage (Lepidoptera: Micropterigidae). Proc Biol Sci. 278(1721):3026–3033. 21367790 10.1098/rspb.2011.0134 PMC3158941

[evab229-B13] Kawahara AY , et al 2019. Phylogenomics reveals the evolutionary timing and pattern of butterflies and moths. Proc Natl Acad Sci U S A. 116(45):22657–22663. 31636187 10.1073/pnas.1907847116 PMC6842621

[evab229-B14] Korf I. 2004. Gene finding in novel genomes. BMC Bioinformatics 5(1):59–59. 15144565 10.1186/1471-2105-5-59 PMC421630

[evab229-B15] Krenn HW. 2010. Feeding mechanisms of adult lepidoptera: structure, function, and evolution of the mouthparts. Annu Rev Entomol. 55:307–327. 19961330 10.1146/annurev-ento-112408-085338 PMC4040413

[evab229-B16] Kristensen NP. 1984. Studies on the morphology and systematics of primitive Lepidoptera (Insecta). Steenstrupia 10:141–191.

[evab229-B17] Kristensen NP , editor. 1999. Lepidoptera, moths and butterflies, Volume 1: evolution, systematics, and biogeography. In: Handbook of Zoology, Volume IV, Arthropoda: insecta, Part 35. Berlin, Germany: Walter de Gruyter. p. 41–50.

[evab229-B18] Kristensen NP , Skalski AW. 1999. Phylogeny and paleontology. In: Kristensen NP, editor. Lepidoptera: moth and butterflies, 1: evolution, systematics and biogeography. Handbook of zoology. Berlin, Germany: Walter de Gruyter. p. 7–25.

[evab229-B19] Kriventseva EV , et al 2019. OrthoDB v10: sampling the diversity of animal, plant, fungal, protist, bacterial and viral genomes for evolutionary and functional annotations of orthologs. Nucleic Acids Res. 47(D1):D807–D811. 30395283 10.1093/nar/gky1053 PMC6323947

[evab229-B20] Laetsch DR , Blaxter ML. 2017. BlobTools: interrogation of genome assemblies. F1000Res. 6:1287.

[evab229-B21] Lee J , et al 2021. The genome sequence of *Samia ricini*, a new model species of lepidopteran insect. Mol Ecol Resour. 21(1):327–339. 32985129 10.1111/1755-0998.13259

[evab229-B22] Luo S , Tang M, Frandsen PB, Stewart RJ, Zhou X. 2018. The genome of an underwater architect, the caddisfly *Stenopsyche tienmushanensis* Hwang (Insecta: Trichoptera). GigaScience 7(12):1–12. 10.1093/gigascience/giy143 PMC6302954 30476205

[evab229-B23] Mackay RJ , Wiggins GB. 1979. Ecological diversity in Trichoptera. Annu Rev Entomol. 24(1):185–208.

[evab229-B24] Mitter C , Davis DR, Cummings MP. 2017. Phylogeny and evolution of Lepidoptera. Annu Rev Entomol. 62:265–283. 27860521 10.1146/annurev-ento-031616-035125

[evab229-B25] Olsen LK , et al 2021. Draft genome assemblies and annotations of *Agrypnia vestita* Walker, and *Hesperophylax magnus* Banks reveal substantial repetitive element expansion in tube case-making Caddisflies (Insecta: Trichoptera). Genome Biol Evol. 13(3):1–7. 10.1093/gbe/evab013 PMC7936034 33501983

[evab229-B26] Ranallo-Benavidez TR , Jaron KS, Schatz MC. 2020. GenomeScope 2.0 and Smudgeplot for reference-free profiling of polyploid genomes. Nat Commun. 11(1):1432. 32188846 10.1038/s41467-020-14998-3 PMC7080791

[evab229-B27] Scoble M. 1992. The Lepidoptera: form, function and diversity. Oxford: Oxford University Press.

[evab229-B28] Seppey M , Manni M, Zdobnov EM. 2019. BUSCO: assessing genome assembly and annotation completeness. Methods Mol Biol. 1962:227–245. 31020564 10.1007/978-1-4939-9173-0_14

[evab229-B29] Smit AFA , Hubley R, Green P. 2013–2015. RepeatMasker Open-4.0 [cited 2021]. Available from: http://www.repeatmasker.org.

[evab229-B34] Thomas JA , Frandsen PB, Prendini E, Zhou X, Holzenthal RW. 2020. A multigene phylogeny and timeline for Trichoptera (Insecta). Syst Entomol. 45(3):670–686.

[evab229-B30] Triant DA , Cinel SD, Kawahara AY. 2018. Lepidoptera genomes: current knowledge, gaps and future directions. Curr Opin Insect Sci. 25:99–105. 29602369 10.1016/j.cois.2017.12.004

[evab229-B31] Trizna M. 2020. assembly_stats 0.1.4. Zenodo. Available from: 10.5281/Zenodo.3968775.

[evab229-B32] Van Nieukerken EJ , et al 2011. Order Lepidoptera Linnaeus, 1758. In: Zhang Z-Q, editor. Animal biodiversity: an outline of higher-level classification and survey of taxonomic richness. Zootaxa 3148(1):212–221. 10.11646/zootaxa.3703.1.1 26146682

[evab229-B33] Whalley PE. 1978. New taxa of fossil and recent Micropterigidae with a discussion of their evolution and a comment on the evolution of Lepidoptera (Insecta). Ann Transvaal Mus. 31(8):71–90.

