# Supplementary material for: First Annotated Genome of a Mandibulate Moth, Neomicropteryx cornuta, Generated Using PacBio HiFi Sequencing
Source: Genome Biol Evol. 2021 Oct 1;13(10):evab229. doi: 10.1093/gbe/evab229 (PMC8557830; doi:10.1093/gbe/evab229)
Supplement: evab229_Supplementary_Data [file evab229_supplementary_data.docx]

**Supplemental Material**

**Supplementary Note 1: DNA extraction, library preparation, and sequencing**

Samples were collected as third to fifth instar larvae of *Neomicropteryx cornuta* from two prefectures in Japan: Kochi Prefecture between October 2-8, 2018 and Ehime Prefecture on February 2, 2019, and each larva was placed individually in a 2 ml cryo-vial. Some larvae were reared in captivity at Ehime University in the lab of YI. Others were flash-frozen on site in a liquid-nitrogen tank and shipped to the Smithsonian National Museum of Natural History (USNM) for deposition in the Biorepository.

A single individual (USNM Barcode: USNMENT01413230; Biorepository Number: AI4GN27), collected from Yasui Keikoku (33.6755, 133.185) on October 3, 2018, was used for genome and transcriptome sequencing.

For genome sequencing, DNA was extracted from the specimen using a Quick-DNA HMW MagBead Kit (Zymo Research). Sequencing libraries were prepared with the SMRTbell Express Template Prep Kit 2.0 (PacBio 101-843-100) using the PacBio low input protocol (DNA sheared to 15kb) for HiFi sequencing followed by AMPure bead cleanup. The genomic library was sequenced on two 30-hour movie 8M SMRT cells in CCS mode on the PacBio Sequel II system at BYU’s DNA Sequencing Center. For transcriptome sequencing, the silk gland and head were dissected and immediately flash frozen in liquid nitrogen. RNA was isolated using Trizol. IsoSeq sequencing library was prepared following the NEBNext Single Cell/Low Input cDNA Synthesis & Amplification Module for the SMRTbell Express Template Prep Kit 2.0 using the IsoSeq express workflow. The resulting library was sequenced on a single Sequel II PacBio SMRT cell for 30 hrs.

**Supplementary Note 2: Contamination-screening using BlobTools**

We screened the final genome assemblies for potential contamination with taxon-annotated GC-coverage (TAGC) plots using BlobTools v1.0 (Laetsch and Blaxter 2017). For this purpose, we mapped all HiFi reads against the final genome assemblies using minimap2 (Li 2018) with parameters *-ax asm20* and sorted the resulting bam file with *samtools sort*. Taxonomic assignment for BlobTools was done with blastn using the following parameters: -task megablast -query neomicropteryx_assembly -db ncbi_nt/nt -outfmt '6 qseqid staxids bitscore std' -num_threads 30 -max_target_seqs 1 -max_hsps 1-e value 1e-25 -out Neomicropteryx_vs_nt. The blobtools module *map2cov* was used to calculate the coverage and *blobtools create* was used to create the blobtools DB. We used the blobtools module *plot* to visualize the DB (Supplementary Figure 1).


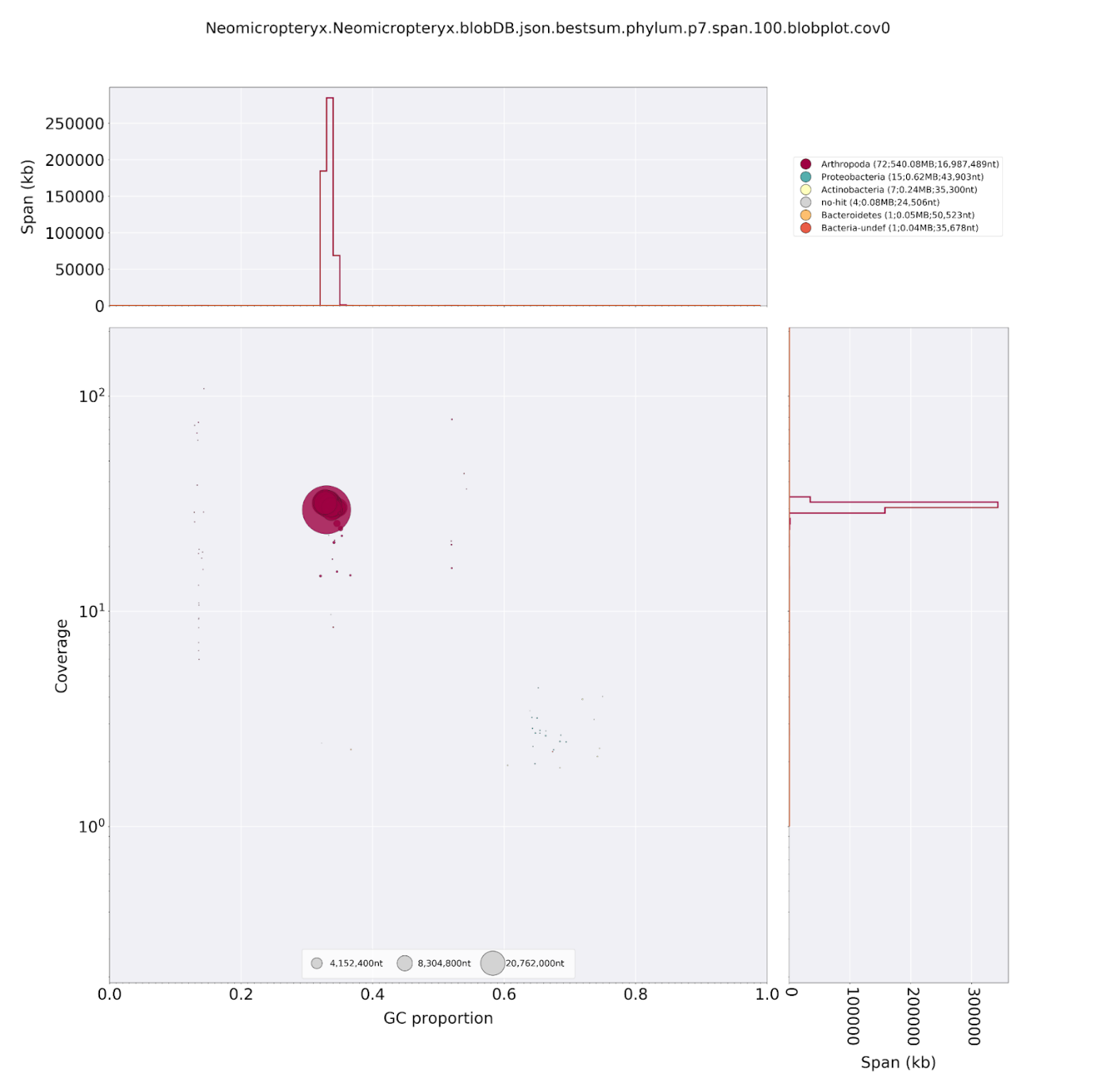


**Supplementary Figure 1.** Taxon-annotated GC-coverage (TAGC) plots for the final genome assemblies of *N. cornuta*. Contigs are represented with circles. Colors indicate the best match to the corresponding taxonomic annotation. The distribution of the total span (kb) of contigs for a given GC proportion or coverage is provided in the upper- and right panels respectively.

**Supplementary Note 3: BUSCO analysis and genome statistics**

We assessed genome quality and completeness with BUSCO v4.1.1 (Seppey, Manni, and Zdobnov 2019) with the insecta_odb10 dataset (https://busco-data.ezlab.org/v4/data/lineages/insecta_odb10.2020-08-05.tar.gz) with options --long -m genome and --offline. The results are summarized in Figure 1 and Table 1. BUSCO v.4.1.1 was also run using the endopterygota_odb10 dataset (https://busco-data.ezlab.org/v4/data/lineages/endopterygota_odb10.2020-08-05.tar.gz). In total, BUSCO detected 97.3% of the Endopterygota core gene collection in the predicted proteins of *N. cornuta* (complete: 96.1%, fragmented: 1.2%).

**Supplementary Table 1.** Assembly statistics for *N. cornuta*. Assembly stats were calculated using the assembly_stats.py function (Mike Trizna, 2020)

sum = 541115538, n = 101, ave = 5357579.58, largest = 83047789

N50 = 16921359, n = 11

N60 = 15429570, n = 14

N70 = 13168023, n = 18

N80 = 11950498, n = 22

N90 = 9924574, n = 27

N100 = 7294, n = 101

N_count = 0

Gaps = 0

**Supplementary Note 4: Genome profiling based on a k-mer distribution-based method**

Before running GenomeScope 2.0 (Ranallo-Benavidez, Jaron, and Schatz 2020), we counted k-mers with jellyfish v2.2.10 (Marçais and Kingsford 2011) using *jellyfish count* -C -s 25556999998 -F 3 and a *k-mer* length of 21 (-m 21) as recommended for most genomes by the authors of GenomeScope2. Using *jellyfish* *histo*, we produced a histogram of k-mer frequencies. We ran GenomeScope 2.0 with the exported *k-mer* count histogram within the online web tool (http://qb.cshl.edu/genomescope/genomescope2.0/) using the following parameters: *k-mer* length = 21 and max kmer coverage = 10000. We conducted a second genome size estimation using backmap.pl v0.3 (Schell et al. 2017) with the options -nq and the sorted .bam files resulting from the re-mapping step. This script uses BEDTools v2.27.1 (https://bedtools.readthedocs.io/en/latest/) to generate a coverage histogram and R v3.5.1 (R Core Team, 2017; https://www.R-project.org/) to plot the coverage distribution. Assuming even sequencing coverage throughout the genome, backmap.pl estimates the genome size by dividing the number of total nucleotides which were mapped to the assembly by the maximum of the per-position coverage frequency distribution.

The estimated genome size for *N. cornuta* resulting from the *k-mer* based method with Genomescope2 was 493,189,997 bp and uniqueness was 73.8% (Supplementary Figure 2). The mapping-based genome size estimation with backmap.pl was 531 Mb (Supplementary Figure 3).

http://qb.cshl.edu/genomescope/genomescope2.0/analysis.php?code=dQ0suZYipzH3vMwr3lV


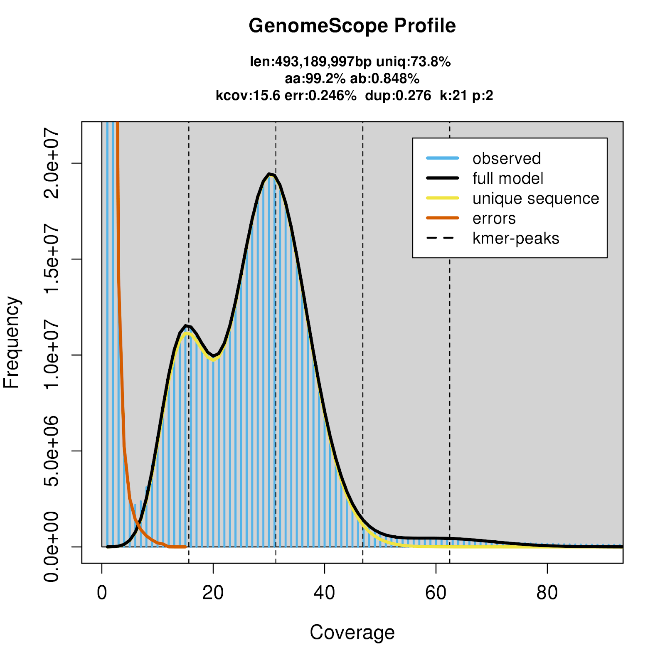

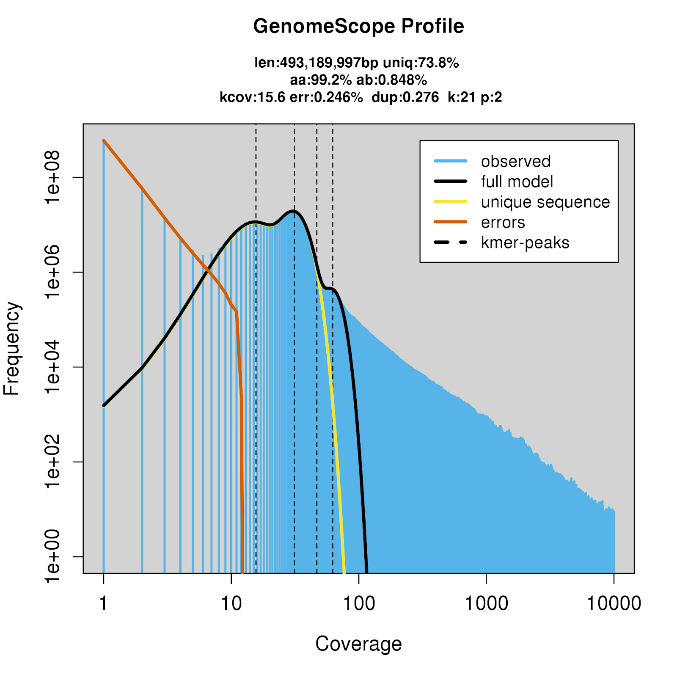


**Supplementary Figure 2.** GenomeScope Profile generated by Genomescope2.


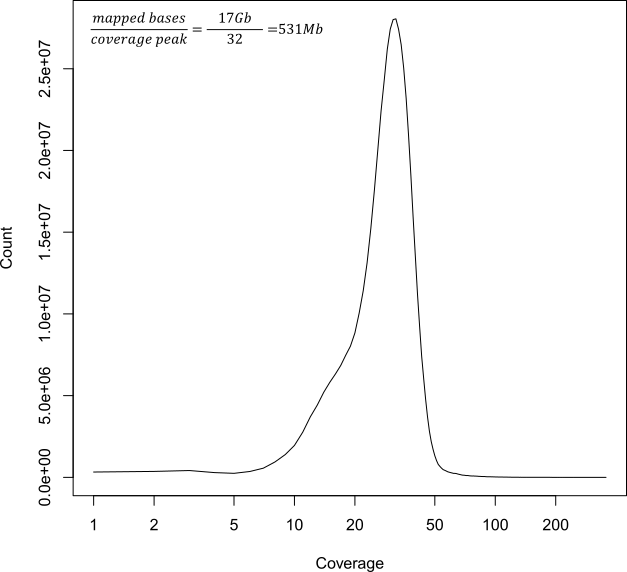


**Supplementary Figure 3.** Mapping-based genome size estimation generated by backmap.pl.

**Supplementary Note 5: Genome assembly annotation**

We annotated the *N. cornuta* genome assembly using MAKER v3.01.03 (Cantarel et al. 2008). We first annotated and masked repeats using the Repbase *Bombyx* repeat library (Bao, Kojima, and Kohany 2015) in RepeatMasker v4.0.9 (Smit, Hubley, and Green, n.d.) in our first run of MAKER together with four additional sources of genetic data: (1) Our *N. cornuta* IsoSeq transcriptome assembly of the silk gland from the same individual, (2) An additional micropterigid transcriptome assembly of *Micropterix calthella* (SRR596161), (3) Protein evidence from *Bombyx mori* (GCF_014905235.1), and (4) Genome annotation from *Bombyx mori*. In order to assemble the transcriptome data from *M. calthella*, we trimmed low quality (<20 quality score, reads <30 length) base calls in trim_galore (http://www.bioinformatics.babraham.ac.uk/projects/trim_galore/) and performed a *de novo* assembly in Trinity v2.8.4 (Haas et al. 2013). We then generated *ab initio* gene predictions using SNAP (Korf 2004). First, we trained a SNAP model with the output from the first round of MAKER. Then, we ran MAKER a second time with the SNAP trained model and the first round of MAKER output to annotate and predict additional genes in the *Neomicropteryx* genome assembly. We again processed the output of this MAKER run through SNAP (second round) and then ran a final (third) round of MAKER to predict genes using the trained SNAP model and the second round of MAKER output. Afterwards, we characterized the gene models from SNAP using ncbi-blast 2.10.1, blastp with -e-value 1e-4, -max_hsps 5, -outfmt 6, and -max_target_seqs 5. Functional annotations were assigned using Blast2GO (Götz et al. 2008).


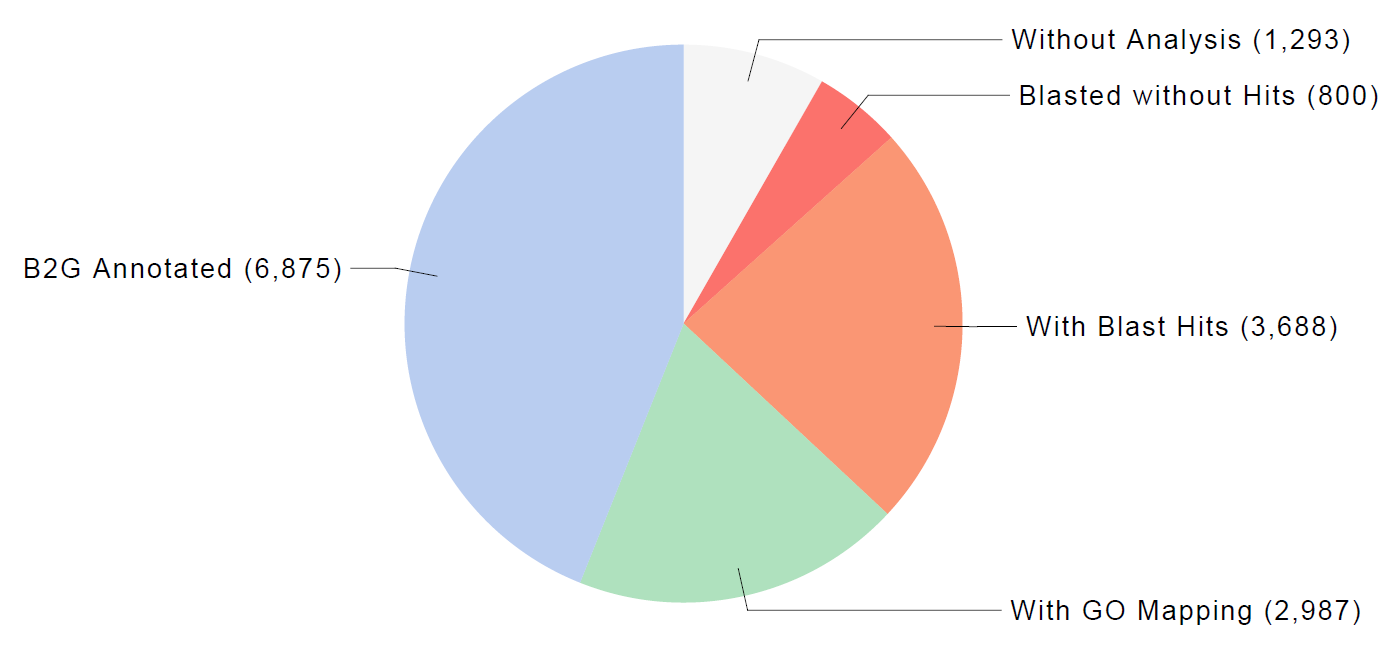


**Supplementary Figure 4.** Blast2Go annotation results for *N. cornuta*. Pie chart showing the percentage of proteins in *N. cornuta* with functional Blast2GO annotations that were verified by BLAST and mapped to GO terms compared to proteins lacking a functional annotation but verified by BLAST and mapped to GO terms or proteins only verified by BLAST, or not verified by BLAST.


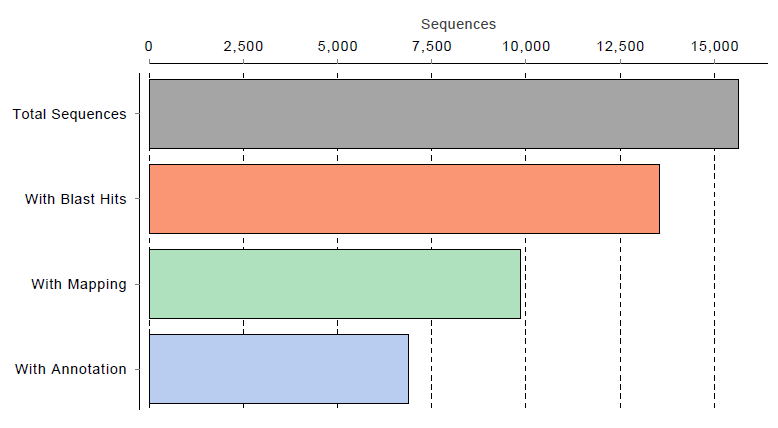


**Supplementary Figure 5.** Blast2Go annotation results for *N. cornuta*. Barplot showing the total number of sequences which obtained results during the different analysis steps (verified by BLAST, mapped to GO terms, and with functional Blast2GO annotations).


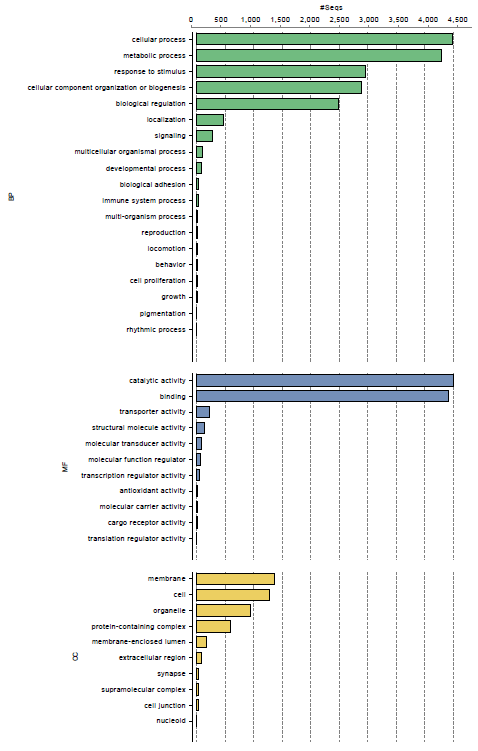


**Supplementary Figure 6.** Blast2GO functional annotation for *N. cornuta*. Barplot showing GO terms characterized by biological process (BP), molecular function (MF), and cellular component (CC).

**Literature Cited**

Bao, Weidong, Kenji K. Kojima, and Oleksiy Kohany. 2015. “Repbase Update, a Database of Repetitive Elements in Eukaryotic Genomes.” *Mobile DNA* 6 (1): 4–9. https://doi.org/10.1186/s13100-015-0041-9.

Cantarel, Brandi L., Ian Korf, Sofia M.C. Robb, Genis Parra, Eric Ross, Barry Moore, Carson Holt, Alejandro Sánchez Alvarado, and Mark Yandell. 2008. “MAKER: An Easy-to-Use Annotation Pipeline Designed for Emerging Model Organism Genomes.” *Genome Research* 18 (1): 188–96. https://doi.org/10.1101/gr.6743907.

Götz, Stefan, Juan Miguel García-Gómez, Javier Terol, Tim D. Williams, Shivashankar H. Nagaraj, María José Nueda, Montserrat Robles, Manuel Talón, Joaquín Dopazo, and Ana Conesa. 2008. “High-Throughput Functional Annotation and Data Mining with the Blast2GO Suite.” *Nucleic Acids Research* 36 (10): 3420–35. https://doi.org/10.1093/nar/gkn176.

Haas, Brian J., Alexie Papanicolaou, Moran Yassour, Manfred Grabherr, Philip D. Blood, Joshua Bowden, Matthew Brian Couger, et al. 2013. “De Novo Transcript Sequence Reconstruction from RNA-Seq Using the Trinity Platform for Reference Generation and Analysis.” *Nature Protocols* 8 (8): 1494–1512. https://doi.org/10.1038/nprot.2013.084.

Korf, Ian. 2004. “Gene Finding in Novel Genomes.” *BMC Bioinformatics* 5: 1–9. https://doi.org/10.1186/1471-2105-5-59.

Laetsch, Dominik R, and Mark L Blaxter. 2017. “BlobTools : Interrogation of Genome Assemblies.” *F1000Res* 1287 (6).

Li, Heng. 2018. “Minimap2: Pairwise Alignment for Nucleotide Sequences.” *Bioinformatics* 34 (18): 3094–3100. https://doi.org/10.1093/bioinformatics/bty191.

Marçais, Guillaume, and Carl Kingsford. 2011. “A Fast, Lock-Free Approach for Efficient Parallel Counting of Occurrences of k-Mers.” *Bioinformatics* 27 (6): 764–70. https://doi.org/10.1093/bioinformatics/btr011.

Ranallo-Benavidez, T. Rhyker, Kamil S. Jaron, and Michael C. Schatz. 2020. “GenomeScope 2.0 and Smudgeplot for Reference-Free Profiling of Polyploid Genomes.” *Nature Communications* 11 (1). https://doi.org/10.1038/s41467-020-14998-3.

Schell, Tilman, Barbara Feldmeyer, Hanno Schmidt, Bastian Greshake, Oliver Tills, Manuela Truebano, Simon D. Rundle, Juraj Paule, Ingo Ebersberger, and Markus Pfenninger. 2017. “An Annotated Draft Genome for Radix Auricularia (Gastropoda, Mollusca).” *Genome Biology and Evolution* 9 (3): 585–92. https://doi.org/10.1093/gbe/evx032.

Seppey, Mathieu, Mosè Manni, and Evgeny M Zdobnov. 2019. “BUSCO: Assessing Genome Assembly and Annotation Completeness.” *Methods in Molecular Biology (Clifton, NJ)* 1962: 227–45.

Smit, AFA, R Hubley, and P Green. n.d. “RepeatMasker Open-4.0 [Cited 2021 ].” *Available from: Http://Www.Repeatmasker.Org*.
